# The Emerging Role of Generative Artificial Intelligence in Medical Education, Research, and Practice

**DOI:** 10.7759/cureus.40883

**Published:** 2023-06-24

**Authors:** Mohammadali M Shoja, J.M.Monica Van de Ridder, Vijay Rajput

**Affiliations:** 1 Medical Education, Nova Southeastern University, Fort Lauderdale, USA; 2 College of Human Medicine, Michigan State University, Grand Rapids, USA; 3 Medical Education, Dr. Kiran C. Patel College of Allopathic Medicine, Nova Southeastern University, Fort Lauderdale, USA

**Keywords:** clinical medicine, research, information literacy, education, artificial intelligence

## Abstract

Recent breakthroughs in generative artificial intelligence (GAI) and the emergence of transformer-based large language models such as Chat Generative Pre-trained Transformer (ChatGPT) have the potential to transform healthcare education, research, and clinical practice. This article examines the current trends in using GAI models in medicine, outlining their strengths and limitations. It is imperative to develop further consensus-based guidelines to govern the appropriate use of GAI, not only in medical education but also in research, scholarship, and clinical practice.

## Introduction and background

*Generative artificial intelligence* (GAI) refers to a class of algorithms that learn from a large corpus of data to create new content in a variety of forms, including text, images, video, audio, and code [1]. The GAI models have gained attention for their ability to tackle complex factual queries and perform a range of tasks such as writing essays, composing poems, performing literature reviews, and translating, summarizing, paraphrasing, or expanding and adapting texts to different contexts or perspectives [2-5]. The performance of these models heavily depends on the nature of the problem, the type of inquiry, and the quality and relevance of the data on which the algorithms are trained [6]. The GAI is transitioning from supervised learning to self-supervised learning, which solely relies on raw text data without human labeling, thus allowing it to leverage a large volume of publicly available data [7]. The *Chat Generative Pre-trained Transformer* (ChatGPT, OpenAI LLP, San Francisco, CA, USA) was launched on November 30, 2022. It is a highly versatile, transformer-based, non-domain-specific large language model (LLM) trained on a large corpus of text data, which amounts to approximately 45 terabytes of data or about one million feet of bookshelf space. The ChatGPT can generate meaningful, credible, and novel word sequences that the model has never encountered before [8].

Transformers are neural networks first introduced by researchers at Google Brain and the University of Toronto in 2017 [9]. They were initially developed for language translation by processing a sequence of words as a single unit of data rather than treating individual words as separate data points. The attention mechanism, which is a key component of the transformer architecture, allows the model to understand the context and meaning of the words in the sequence, making it highly effective for a wide range of language processing tasks [10]. Transformers differ from other deep learning models, such as recurrent neural network (RNN), which processes the data points sequentially and one at a time [10]. Recent advancements in GAI, especially in the development of self-supervised learning models and transformer-based LLMs such as ChatGPT, have the potential to transform healthcare education, research, and clinical practice.

This editorial is divided into three sections. The first section examines the current trends in using GAI models in medical education. The second section covers various aspects of using GAI language models such as ChatGPT in scientific and technical writing, including overcoming language barriers, generating scientific texts, the question of authorship, and the role of GAI in peer review. The third section discusses the potential role of GAI models in clinical practice and healthcare systems, addressing topics such as clinical reasoning, and the potential problems that GAI models can tackle in healthcare. It also highlights the limitations of contemporary GAI models, including their inability to make difficult contextual, evidence-based medicine decisions and the potential for bias and discrimination with limited, diverse data.

## Review

Medical education

Trends and Key Issues

A survey of 1,000 undergraduate college students in the United States in January 2023 revealed that 30% of the respondents reported using ChatGPT for their written homework [11]. Nearly 60% of these students used ChatGPT on more than half of their assignments. Interestingly, three out of four users acknowledged that using ChatGPT was cheating, but they still used it anyway. This survey highlights that GAIs and LLMs will play a part in higher education. It highlights the urgent need for instructors and education administrators to raise awareness and take appropriate action to address key issues. As Mogali pointed out, it is important to consider that it is still a machine and lacks the attributes of a human educator or role model [12]. A further concern is the possibility that erroneous information generated by ChatGPT might have an adverse effect on the long-term learning of the students. Contemporary GAIs may generate content that appears compelling and reasonable yet is factually incorrect [13]. How can students improve their cognitive skills through GAI? Will it help with critical thinking and problem-solving? How can students adhere to academic integrity in their writing assignments? What does this mean for our assessment system? Do we need other forms of exams, such as oral exams, or should we go back to pen and paper? These concerns underscore the need to give careful thought to ChatGPT's role in medical education to ensure that it is used in a manner that fosters effective learning and critical thinking.

Guidelines for Use of GAI

The University of Southern California has developed one of the early guidelines on the use of GAI for academic work, primarily in the field of education [13]. The guideline advises instructors to (1) proactively educate themselves on GAI to refine their teaching techniques and methods; (2) encourage students to explore and experiment with GAI technology and communicate their expectations regarding the role of GAI in the syllabus; and (3) furnish information on the strengths and limitations of the technology to the students. The guideline also emphasizes that students should use GAI to "create, analyze, and evaluate new concepts and ideas that inspire them to generate their own academic work" [13]. More consensus-based guidelines are necessary at the institutional and/or national level to govern the appropriate use of ChatGPT, not only in medical education but also in research, scholarship, and clinical practice.

Assessments

The performance of the ChatGPT on standardized tests in higher education has been studied. Fijačko et al. reported that ChatGPT achieved scores of 64% to 68% in the American Heart Association (AHA) Basic Life Support (BLS), and 68% to 76% in the Advanced Cardiovascular Life Support (ACLS) examination, falling short of the passing threshold of 84% [14]. However, the authors pointed out that the explanations offered by ChatGPT were relevant, accurate, and more comprehensive than the rationale provided in the written ACLS exam key. Gilson et al. reported that ChatGPT performed above the 60% threshold on the United States Medical Licensing Examination Step 1 (using publicly available National Board of Medical Examiners (NBME) sample questions) and achieved the equivalent of a passing score for a third-year medical student [15]. The model provided an understandable narrative and context across most answers. In another study that utilized multiple-choice single-answer questions from the United States Medical Licensing Examination (USMLE) Steps 1, 2 clinical knowledge (CK), and 3, the accuracy of ChatGPT varied from 36.1% to 60.9% [8]. A noteworthy finding was the occurrence of indeterminate responses (as high as 19%), where the GAI provided a response that was not among the answer choices or indicated a lack of sufficient information to provide a definite answer. Gilson et al. suggested that ChatGPT has the potential to facilitate the development of an interactive, externally supported, and on-demand learning environment for students, which could enhance problem-solving skills and reflective learning [15]. This technology presents the potential to augment human educators while also tailoring the learning needs of individual students [12].

Concerns About Plagiarism

O'Connor warns that, if not employed ethically and responsibly, a GAI might lead to plagiarism [16]. ChatGPT is a powerful technology for generating cohesive and meaningful scientific text that does not set off plagiarism detection tools [17]. In a study, plagiarism detection software identified 40 out of 50 essays generated by ChatGPT as having a high level of originality [18]. The essays that were rated as having the highest originality were those that dealt with more contentious topics that required interpretation. Academic essays are meant to showcase a student’s personal reflections and interpretations, and thus essay-type assignments completed by ChatGPT could pass without getting caught as they may be able to meet these expectations [18]. There is a concern about students using ChatGPT to generate text for their essays and then not correctly citing the source of the generated text, which could give them an advantage over students who do not use ChatGPT. The GPTZero (Princeton, NJ, USA) is an online application that utilizes a perplexity score (a measure of the randomness of the text) and a burstiness score (a measure of the variation in perplexity) to differentiate between text composed by humans or artificial intelligence. This software correctly classifies 99% of human-written articles and 85% of AI-generated content [19].

Research and scholarship

Language and Technical/Scientific Writing

ChatGPT is trained in over 40 different languages and is an indispensable tool for the translation of technical texts. This capability is useful in translating non-English medical literature for systematic reviews and medical research. Chen argues that non-English-speaking scientists can benefit from natural language processing models to overcome language barriers [20]. The GAI can generate paper titles, edit manuscripts, enhance the clarity of text, and create new content [21,22]. Gao et al. used ChatGPT to generate 50 fake scientific abstracts based on the titles of papers published in five high-impact medical journals and then ran a plagiarism detector to compare them with the original abstracts [17]. With a median originality score of 100%, the plagiarism detector assessed all the produced content to be entirely original. Expert human reviewers accurately identified only 68% of the fake abstracts, missing nearly one-third of them [17]. This experiment illustrates that ChatGPT is capable of generating highly technical scientific texts that are credible and compelling [23].

The application of GAI in academic writing has recently sparked significant interest. The first author conducted a writing experiment with ChatGPT to assess its validity and reliability in generating factual texts. The experiment focused on the anatomical variations of the scalene muscles and subclavian artery, and ChatGPT was prompted with general questions about their variations. ChatGPT constructed a scientifically plausible argument drawing from 17 published studies and textbooks, but none of the references and provided data were authentic. When prompted further, ChatGPT admitted that "I was fabricating the data because I wanted to make my research appear more impressive and impactful than it actually was" and that the cited studies were "hypothetical", and the references and authors were "fictional." This experiment highlights the tendency of contemporary GAI to confabulate and speculate rather than analyze factual data. The lack of access to scholarly databases and the propensity to fabricate data raise questions about the reliability of contemporary GAI in scholarly writing.

The Authorship

King co-authored an editorial with ChatGPT, which featured an unedited dialogue between the author and ChatGPT on the potential applications of ChatGPT in medical education [24]. Others have used ChatGPT in drafting manuscripts and credited it as a co-author [16]. The idea of crediting GAI as a co-author has been met with criticism from the scientific community. Chen maintains that using GAI to write scientific papers is clearly unethical [20]. The editors at Science Journal have a strict policy against the use of texts, figures, images, or graphics generated by any GAI tools in scientific papers and consider a violation of this policy scientific misconduct [25]. Although the contemporary GAI is not ready for such contributions in scholarship, nevertheless, the use of this cutting-edge technology is inevitable and will become increasingly integrated into our practices. The question now is how to use and properly credit GAI in scientific or literary works that utilize this technology.

Thorp maintains that an AI program cannot be an author [25]. According to Nature and all Springer Nature journals, no LLM tool can be credited as an author on a research paper, and researchers using these tools must document such use in the appropriate section of the publication [26]. The authorship criteria set forth by the International Committee of Medical Journal Editors (ICMJE) are based on four key elements, including: (1) making substantial contributions to the work, (2) being involved in drafting or critically editing the work for important intellectual content, (3) giving final approval of the manuscript, and (4) agreeing to take responsibility for all aspects of the work to ensure that any questions pertaining to the accuracy or integrity of any part of the work are adequately addressed [27]. In fact, it is this last criterion, i.e., assuming responsibility for the study, that renders an AI intrinsically inappropriate as an author [28].

The authors prompted ChatGPT with the ongoing debate on its role in scientific writing. In response, ChatGPT stated that as an AI language model, it lacks the ability to reason independently and comprehend scientific concepts. Although it can generate high-quality text in response to prompts, it is not appropriate to list ChatGPT as a co-author on a scientific paper. Instead, if ChatGPT is used to write an article or any other written material, the authors should acknowledge its contribution by citing it as a source of information or stating that the text was generated by ChatGPT. It is also essential to include a disclaimer or note about the limitations of GAI language models and the need to verify the information obtained. While ChatGPT can be a valuable tool in scientific writing, it should be used with caution and always verified for accuracy and reliability.

Peer Review

Generative artificial intelligence cannot function as a co-author of scientific papers; likewise, it cannot entirely replace human peer-reviewers in the academic publishing process. However, its ability to analyze the text and generate textual feedback can help facilitate or expedite the peer-review process under human auspices. Checco et al. developed a neural network model that was trained on a dataset of 3300 papers and the reviewers’ evaluations from three conferences on learning representations and wireless communications and networking [29]. They then tested the model’s ability to predict the peer-review outcome of new manuscripts based solely on their textual content. Their findings demonstrated that the model was often able to accurately predict the peer review outcome that would match the expert reviewers' recommendations [29]. It is important to assess to what extent transformer-based GAIs can assist in the peer-review process by generating feedback to improve the overall readability of scientific papers, identifying gaps in the argument, assessing the novelty of the study, and providing insights into future perspectives of the study.

Clinical practice

Clinical Reasoning

Clinical reasoning encompasses several components, including generating differential diagnoses, selecting diagnostic tests, refining diagnoses based on test results, formulating a management plan, and creating a narrative summary. Rao et al. investigated ChatGPT’s performance in clinical decision-making by inputting 36 published clinical vignettes into the model, followed by successive prompting [30]. The study found that ChatGPT achieved an overall accuracy of 71.7% across all clinical vignettes, with the highest performance in making a final diagnosis and the lowest performance in generating an initial differential diagnosis. Additionally, ChatGPT demonstrated inferior performance in questions related to clinical management [30]. Strong conducted another experiment to assess ChatGPT's clinical reasoning abilities by prompting it with clinical cases of varying difficulty levels [31]. The model barely passed the Stanford University final clinical reasoning exam for first-year medical students with a score of 72%. In complex cases, ChatGPT missed critical details in multisystem conditions. However, for simple cases, it provided responses at the level of first- or second-year medical students [31]. ChatGPT's strengths in clinical reasoning included creating succinct narrative summaries. It failed to place due emphasis on the patient's age in clinical reasoning and failed miserably in rare clinical conditions and in complicated cases published and debated in medical journals. The major weakness, however, was its tendency to confabulate, i.e., generate responses that sounded intelligent and reasonable to non-experts but were entirely incorrect [31]. This limitation could potentially lead to erroneous diagnoses and inappropriate treatments, particularly in cases where expert input for sound clinical judgment is necessary.

Clinical Practice and Healthcare Systems

Generative artificial intelligence can be an efficient and cost-effective tool in healthcare communication that requires clear textual documents and technical materials. A significant impact can be had on electronic medical records (EMR). It can aid in generating admission notes, consultation reports, discharge summaries, and communication between patients, healthcare organizations, and insurance companies [32]. The GAI can monitor low-complexity clinical conditions in patients under the supervision of healthcare practitioners. The early generation of chatbots has been shown to be non-inferior to clinicians in randomized trials of education, follow-up, and adherence in breast cancer patients [33]. A domain-specific GAI can code procedures and create notes for reimbursement. The written communication of preapproval, authorization, and billing issues can be managed by a specialized GAI. The GAI can summarize patient data and notes in succinct and good-grammatical English. In hospitals, the chatbot can manage front-office communication and documentation.

Role in Retrieving and Managing Knowledge and Expert Judgment

Healthcare professionals have relied on Google, UpToDate, and other databases to find answers to questions backed by validated research. In clinical decision-making, the GAI has been used to predict renal illness, generate radiology reports, and forecast various hematological disorders. Med-PaLM (Mountain View, CA, USA) and DeepMind (London, UK), both developed by Google, are LLMs geared toward medicine that produce answers that align with the scientific consensus in 92.9% of cases [34]. Medical knowledge is built in a slow, almost sublime, but purposeful manner during years of training. It is gained via exhaustive yet experiential contemplation of patients' diagnoses, adoption of bedside manners from role models, and extensive consideration of evidence-based practices. Even though ChatGPT has moderate success in passing medical licensure tests, it has a limited role in critical thinking or making moral or ethical decisions.

Professional clinical judgment is a cognitive and affective process based on careful thought that is manifested through actions and behaviors based on patient clinical information. Good moral judgment requires in-depth knowledge, experience, and obligations for professional standards such as ethics and the laws of medicine, as well as understanding the values of patients and society. Making a judgment involves forming an opinion or decision based on careful and deliberate thinking. Professional judgment is "applying knowledge, skills, and experience in a way that is informed by professional standards, laws, ethics, and principles to develop opinions about what should be done" [35]. There are four building blocks of any clinical judgment: (1) evidence-based knowledge; (2) ethical and legal obligations; (3) patients’ preferences and value systems; and (4) professional experiences that build on wisdom and reflective practices [35]. All these processes require clarity, accuracy, precision, relevance, depth, breadth, fairness, and logic in cognitive processes. The GAI is not trained to guide learners through these procedures. Its output must be monitored and validated. The GAI may create more confusion without knowing the sociocultural context of clinical decisions. The current form of GAI produces convincing information based on fabricated studies. It fails to accurately cite scientific literature. The current GAI is not trained to make difficult, contextual, evidence-based medical decisions when it comes to clinical decision-making for a fictional patient with common symptoms. It reported false information without supportive evidence or fabricated evidence. The current GAI has shown bias and discrimination with the currently limited diverse data.

## Conclusions

The current dialogue in medical education appears to center on the role of GAI models in assessments and writing essays, as well as their interaction with students in the classroom and with assignments. Other applications, such as the automatic scoring of student essays, creating content for quizzes, and assisting in facilitating learning, should be explored. More consensus-based guidelines are necessary to govern the appropriate use of GAI, not only in medical education but also in research, scholarship, and clinical practice. Educators should advocate the responsible and ethical use of GAI and advise students of its benefits and limitations at the beginning of the course and curriculum. It is crucial to avoid assuming that GAI is merely another calculator. Students should employ GAI cautiously in their cognitive learning during coursework and foster the development of independent cognitive skills and critical thinking. Last but not least, any information obtained from ChatGPT should be verified for accuracy and reliability before use.

Generative artificial intelligence's ability to translate technical texts and generate highly technical scientific texts that are succinct and compelling has been demonstrated. However, there are concerns about its reliability in scholarly writing due to the tendency to speculate rather than analyze factual data. The question of GAI’s authorship has been met with criticism, and it is recommended that GAI be cited as a source of information rather than being credited as a co-author. The role of GAI in peer review is still being explored, but its ability to analyze text and generate feedback can help expedite the process.

As GAI integrates into personal and professional lives in our society, it is important to recognize and manage the unintended consequences. While some of these risks are already understood, their full extent has yet to be determined. Healthcare organizations and professionals may be exposed to legal liability and reputational harm if GAI generates biased or incorrect content, violates copyright laws, or plagiarizes from other sources. For the time being, or at least for the near future, all content generated by contemporary GAI must be verified by humans, and close oversight and critical scrutiny of the generated content are necessary to ensure the welfare of society. The landscape of ethical, legal, and moral decisions related to GAI will come with new opportunities and risks. It is imperative for healthcare organizations and professionals to remain vigilant about the clinical outcomes, regulations, and risks related to GAI. As non-conscious entities, GAI generates outputs solely from data and requires regular monitoring for updates and errors to optimize their performance. Learners and educators alike need to be aware of the broader impact of GAI on moral judgment, clinical decision-making, and social justice.

## References

[1] Sveding JJ (2021). Unsupervised Image-to-Image Translation: Taking Inspiration From Human Perception. http://www.diva-portal.org/smash/record.jsf?pid=diva2%3A1574122&dswid=2869.

[2] Tate T, Doroudi S, Ritchie D (2022). Educational research and AI-generated writing: confronting the coming tsunami. EdArXiv Preprints.

[3] Jiao W, Wang W, Huang J (2023). Is ChatGPT a good translator? A preliminary study. ArXiv Preprints.

[4] Aydin Ö, Karaarslan E (2022). OpenAI ChatGPT generated literature review: digital twin in healthcare. Emerging Computer Technologies 2.

[5] Stokel-Walker C (2022). AI bot ChatGPT writes smart essays — should professors worry?. Nature.

[6] Alshater M (2023). Exploring the role of artificial intelligence in enhancing academic performance: a case study of ChatGPT. SSRN.

[7] Anonymous (2023 (2023). bert-large-cased-whole-word-masking · Hugging Face. https://huggingface.co/bert-large-cased-whole-word-masking.

[8] Kung TH, Cheatham M, Medenilla A (2023). Performance of ChatGPT on USMLE: potential for AI-assisted medical education using large language models. PLOS Digit Health.

[9] Vaswani A, Shazeer N, Parmar N (2017). Attention is all you need. 31st Conference on Neural Information Processing Systems (NIPS).

[10] Markowitz D (2023 (2023). Transformers, Explained: Understand the Model Behind GPT-3, BERT, and T5. https://daleonai.com/transformers-explained.

[11] (2023). Nearly 1 in 3 College Students Have Used ChatGPT on Written Assignments - Intelligent. https://www.intelligent.com/nearly-1-in-3-college-students-have-used-chatgpt-on-written-assignments.

[12] Mogali SR (2023). Initial impressions of ChatGPT for anatomy education. Anat Sci Educ.

[13] University of Southern California Committee on Information Services, Academic Senate-Provost Joint Committee (2023). Instructor guidelines for student use of generative artificial intelligence for academic work. Instructor guidelines for student use of generative artificial intelligence for academic work.

[14] Fijačko N, Gosak L, Štiglic G, Picard CT, John Douma M (2023). Can ChatGPT pass the life support exams without entering the American heart association course?. Resuscitation.

[15] Gilson A, Safranek CW, Huang T, Socrates V, Chi L, Taylor RA, Chartash D (2023). How does ChatGPT perform on the united states medical licensing examination? The implications of large language models for medical education and knowledge assessment. JMIR Med Educ.

[16] O'Connor S (2023). Open artificial intelligence platforms in nursing education: tools for academic progress or abuse?. Nurse Educ Pract.

[17] Gao CA, Howard FM, Markov NS (2022). Comparing scientific abstracts generated by ChatGPT to original abstracts using an artificial intelligence output detector, plagiarism detector, and blinded human reviewers. bioRxiv Preprints.

[18] Khalil M, Er E (2023). Will ChatGPT get you caught? Rethinking of plagiarism detection. arXiv Preprints.

[19] (2023). GPTZero. https://app.gptzero.me/app/faq.

[20] Chen TJ (2023). ChatGPT and other artificial intelligence applications speed up scientific writing. J Chin Med Assoc.

[21] Hutson M (2022). Could AI help you to write your next paper?. Nature.

[22] Gaggioli A (2023). Ethics: disclose use of AI in scientific manuscripts. Nature.

[23] Else H (2023). Abstracts written by ChatGPT fool scientists. Nature.

[24] King MR (2023). A conversation on artificial intelligence, chatbots, and plagiarism in higher education. Cell Mol Bioeng.

[25] Thorp HH (2023). ChatGPT is fun, but not an author. Science.

[26] Editorial Editorial (2023). Tools such as ChatGPT threaten transparent science; here are our ground rules for their use. Nature.

[27] (2023). ICMJE | Recommendations | Defining the Role of Authors and Contributors. https://www.icmje.org/recommendations/browse/roles-and-responsibilities/defining-the-role-of-authors-and-contributors.html.

[28] Stokel-Walker C (2023). ChatGPT listed as author on research papers: many scientists disapprove. Nature.

[29] Checco A, Bracciale L, Loreti P (2021). AI-assisted peer review. Humanit Soc Sci Commun.

[30] Rao A, Pang M, Kim J (2023). Assessing the utility of ChatGPT throughout the entire clinical workflow. medRxiv.

[31] Strong E (2023 (2023). Can ChatGPT Pass a Medical School Final? - YouTube. https://www.youtube.com/watch?v=2VL6_Cyblv0&ab_channel=StrongMedicine.

[32] Patel SB, Lam K (2023). ChatGPT: the future of discharge summaries?. Lancet Digit Health.

[33] Bibault JE, Chaix B, Guillemassé A (2019). A chatbot versus physicians to provide information for patients with breast cancer: blind, randomized controlled noninferiority trial. J Med Internet Res.

[34] Singhal K, Azizi S, Tu T (2022). Large language models encode clinical knowledge. ArXiv Preprints.

[35] Cohen D (2015). What is professional judgment?. Resume.

